# Magnetic Hyperbolic Metasurface: Concept, Design, and Applications

**DOI:** 10.1002/advs.201801495

**Published:** 2018-11-12

**Authors:** Yihao Yang, Pengfei Qin, Bin Zheng, Lian Shen, Huaping Wang, Zuojia Wang, Erping Li, Ranjan Singh, Hongsheng Chen

**Affiliations:** ^1^ State Key Laboratory of Modern Optical Instrumentation and The Electromagnetics Academy at Zhejiang University Zhejiang University Hangzhou 310027 China; ^2^ Key Laboratory of Micro‐Nano Electronics and Smart System of Zhejiang Province College of Information Science and Electronic Engineering Zhejiang University Hangzhou 310027 China; ^3^ Division of Physics and Applied Physics School of Physical and Mathematical Sciences Nanyang Technological University 21 Nanyang Link Singapore 637371 Singapore; ^4^ Centre for Disruptive Photonic Technologies The Photonics Institute Nanyang Technological University 50 Nanyang Avenue Singapore 639798 Singapore; ^5^ Institute of Marine Electronics Engineering Ocean College Zhejiang University Hangzhou 310058 China; ^6^ School of Information Science and Engineering Shandong University Jinan 250100 China

**Keywords:** hyperbolic metasurfaces, magnetic surface plasmons, metamaterials

## Abstract

A fundamental cornerstone in nanophotonics is the ability to achieve hyperbolic dispersion of surface plasmons, which shows excellent potentials in many unique applications, such as near‐field heat transport, planar hyperlens, strongly enhanced spontaneous emission, and so forth. The hyperbolic metasurfaces with such an ability, however, are currently restricted to electric hyperbolic metasurface paradigm, and realization of magnetic hyperbolic metasurfaces remains elusive despite the importance of manipulating magnetic surface plasmons (MSPs) at subwavelength scale. Here, magnetic hyperbolic metasurfaces are proposed and designed, on which diffraction‐free propagation, anomalous diffraction, negative refraction, and frequency‐dependent strong spatial distributions of the MSPs in the hyperbolic regime are experimentally observed at microwave frequencies. The findings can be applied to manipulate MSPs and design planarized devices for near‐field focusing, imaging, and spatial multiplexers. This concept is also generalizable to terahertz and optical frequencies and inspires novel quantum optical apparatuses with strong magnetic light–matter interactions.

Metasurfaces,^1^, ^2^, ^3^, ^4^, ^5^, ^6^, ^7^, ^8^ with an intrinsically planar nature and subwavelength thickness, provide us with unconventional methodologies to not only mold the flow of propagating waves but also manipulate the near‐field waves. Hyperbolic metasurfaces^6^, ^9^, ^10^, ^11^, ^12^, ^13^, ^14^, ^15^, ^16^, ^17^, ^18^ is a class of metasurfaces with an in‐plane hyperbolic dispersion on which in‐plane surface plasmons^19^, ^20^, ^21^, ^22^ (SPs) propagate with a concave phase front. Researchers have reported many interesting phenomena occurring on hyperbolic metasurfaces, such as negative refraction,^10^, ^11^ nondiffraction propagation,^6^, ^10^, ^11^ anomalous diffraction,^10^ the plasmonic spin Hall effect,^11^ strong spatial localization,^23^ and large local density states.^16^, ^23^ In comparison with traditional bulky metamaterials,^24^, ^25^, ^26^, ^27^, ^28^, ^29^, ^30^, ^31^, ^32^ planar metasurfaces exhibit lower propagation loss and better compatibility with integrated metamaterial circuits and optoelectronic components.^11^, ^18^ Therefore, hyperbolic metasurfaces hold a promising future for designing applications oriented near‐field focusing and imaging devices,^6^, ^18^ enhanced spontaneous emission,^16^, ^23^ planar hyperlenses,^9^ near‐field heat transport,^6^ quantum optics, and information.^11^

It is well known that at the interface between two media with opposite permittivity/permeability, there exists an electromagnetic (EM) surface mode, which is the electric/magnetic SP.^19^, ^20^, ^21^, ^22^, ^33^, ^34^, ^35^, ^36^, ^37^, ^38^ It has been demonstrated that noble metals, e.g., silver and gold, can support electric SPs at optical frequencies (**Figure** **1**a) and that a split‐ring resonator (SRR) chain with a strong magnetic response can support magnetic SPs (MSPs)^34^, ^37^ (Figure 1b). However, the majority of previous works on electric hyperbolic metasurfaces,^11^, ^13^, ^14^, ^15^, ^18^, ^23^ where the electric SPs propagate in a convergent fashion due to anisotropic electric excitations consisting of oscillations in the density of electrons in metals^11^ or graphene^23^ (Figure 1c). The experimental demonstration of the magnetic hyperbolic metasurfaces remains elusive despite the importance of manipulating MSPs at subwavelength scale.

**Figure 1:**
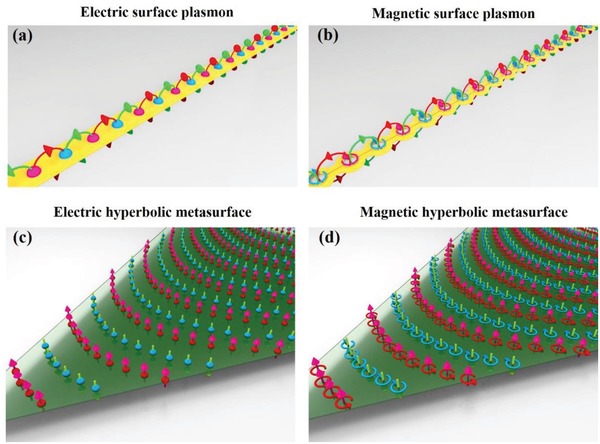
From electric/magnetic SPs to electric/magnetic hyperbolic metasurfaces. a) Electric SPs. Noble metals, such as silver and gold, can support electric SPs at optical frequencies. b) Magnetic SPs. An SRR chain with a strong magnetic response can support magnetic SPs. c) Electric hyperbolic metasurface. On an electric hyperbolic metasurface, the electric SPs propagate with a concave wavefront due to anisotropic electric excitations consisting of oscillations in the density of electrons in the metal/graphene. Here, the blue and the red spherical particles represent positive/negative charges, respectively; the green and the pink arrows represent electric fields with opposite phases. d) MHMS. On the MHMS, the MSPs travel with a concave wavefront, stemming from anisotropic magnetic excitations consisting of coupled magnetic dipoles. Here, the blue and the red rings represent negative/positive circle surface/displacement currents providing strong magnetic dipole moments, respectively; the green and the pink arrows represent magnetic fields with opposite phases.

To overcome this challenge, we propose the concept of a magnetic hyperbolic metasurface (MHMS), where MSPs^34^, ^35^, ^36^, ^37^, ^38^ travel in a convergent manner, stemming from anisotropic magnetic excitations, which can be achieved with coupled magnetic dipoles provided, in practice, by circular surface/displacement electric currents (Figure 1d). As a proof of concept, we designed, fabricated, and experimentally characterized an ultrathin MHMS based on double‐slit SRR^39^ arrays. We directly observe diffraction‐free propagation, anomalous diffraction, all‐angle negative refraction, and frequency‐dependent strong spatial distribution of the MSPs in the hyperbolic regime. Our findings can be used to manipulate MSPs and to devise planarized focusing and imaging devices and spatial multiplexers. Although this concept is verified in the microwave region, this concept is very general and could be applied to terahertz or optical frequencies, thus inspiring novel quantum optical apparatuses with strong magnetic light–matter interactions.

The proposed practical MHMS is shown in **Figure** **2**a, which consists of arrays of double‐slit SRRs. The inset of the figure shows the details of the SRRs, where the golden and brown regions indicate a copper substrate and a dielectric substrate, respectively. Here, *p* = 6 mm, *a* = 5.5 mm, *g* = 0.25 mm, *w* = 0.5 mm, *h* = 1 mm, and the thickness of the copper film is 0.035 mm. The relative permittivity of the substrate is 2.55 + 0.002i at 10.0 GHz. The substrate, which is used to support the copper film and can be removed, has a slight impact on the EM properties of the MHMS. Therefore, the thickness of the metasurface can be extremely thin, e.g., 1/10^4^ times the operational wavelength.

**Figure 2:**
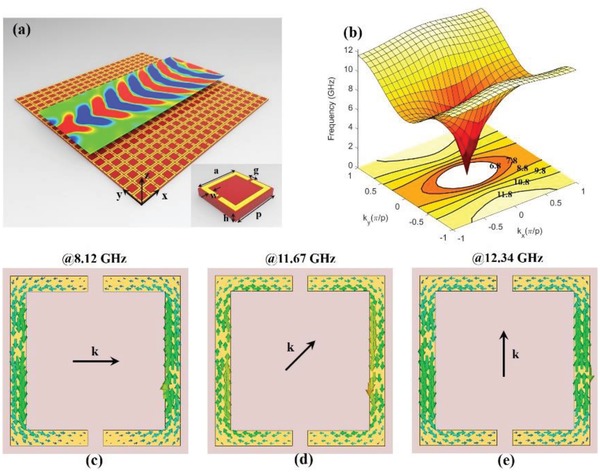
Designed MHMS. a) The designed MHMS consists of double‐slit SRR arrays. The inset shows the details of the SRRs, where the golden and brown regions indicate copper and the substrate, respectively. Here, *p* = 6 mm, *a* = 5.5 mm, *g* = 0.5 mm, *w* = 0.5 mm, *h* = 1 mm, and the thickness of the copper film is 0.035 mm. The permittivity of the substrate is 2.55 + 0.002i at 10.0 GHz. The present metasurface supports hyperbolic SPs at 10.8 GHz. b) 3D perspective view and contours of the dispersion in the first Brillouin zone in the k‐vector space of the MHMS. From 9.8 to 11.8 GHz, the EFCs are hyperbolic. c–e) Surface currents on the present MHMS at several wavevectors at the boundaries of the FBZ. The cut‐off frequency depends on the direction of the incident wavevectors.

By employing the eigenvalue module of the commercial software Computer Simulation Technology (CST) Microwave Studio, we obtain equal‐frequency contours (EFCs) in the first Brillouin zone (FBZ) of the present metasurface (Figure 2b). It is shown that the shapes of the EFCs gradually transition from a closed elliptical ring to an open hyperbolic curve. A transition point occurs at 9.8 GHz; the hyperbolic regime arises from 9.8 to 11.8 GHz. In Figure 2c–e, we slowly rotate the propagation direction of the MSPs and find that the cut‐off frequencies at the boundaries of the FBZ also change accordingly. When the wavevectors are along the horizontal, diagonal, and vertical directions, the cut‐off frequencies are 8.12, 11.67, and 12.34 GHz, respectively. Besides, the SRRs at these cut‐off frequencies show strong magnetic responses. Therefore, the anisotropic magnetic responses of the MHMS stem from the anisotropic distribution of the coupled magnetic dipoles provided by the circular surface currents on the double‐slit SRRs. We also perform the multipolar analysis which reveals that in the hyperbolic regime, the magnetic dipole moment is dominant (see the Supporting Information). Besides, we establish an equivalent circuit model to explain the transition of EFCs of the represent metasurface in the Supporting Information.

To visualize the magnetic field distribution over the metasurface, we perform full‐wave simulations in the time domain module of the CST by considering a practical metasurface that consists of arrays of double‐slit SRRs. In the simulations, we place an electric dipole moment at the slit of an SRR to excite the EM mode of interest. 3D full‐wave simulation results are shown in **Figure** **3**. We can see that at 7.8 GHz, the EFC is an ellipse and the MSPs with a convex wavefront can propagate along all directions on the metasurface. At 8.8 GHz, the EFC becomes extremely anisotropic. Although the MSPs still propagate in a normal diffraction manner, their radiation angle decreases. At the transition point, namely, 9.8 GHz, the EFC consists of two flat lines, and the propagation of the MSPs becomes nondiffracting. From 10.8 to 11.8 GHz, the EFCs change to hyperbolas, the wavefronts of the MSPs are now concave, and the MSPs travel with anomalous diffraction.

**Figure 3:**
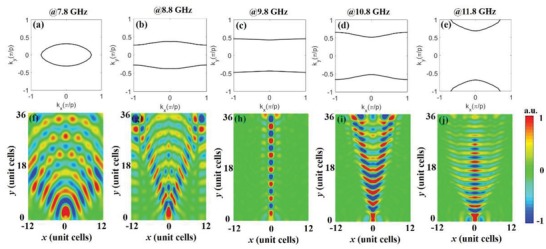
Simulated EFCs and *H_z_* field distributions at five different frequencies. The top row of panels represents the EFCs in the first Brillouin zone of the present MHMS at a) 7.8 GHz, b) 8.8 GHz, c) 9.8 GHz, d) 10.8 GHz, and e) 11.8 GHz. The bottom row of panels represents the *H_z_* field distributions at a plane 6 mm above the metasurface that was excited by an electric dipole at the slit of an SRR at f) 7.8 GHz, g) 8.8 GHz, h) 9.8 GHz, i) 10.8 GHz, and j) 11.8 GHz.

We fabricate the designed MHMS with the aid of standard commercial printed circuit board technology. Experimental measurements are carried out to characterize the EM properties of the metasurface. In the experiment, we use an electric dipole antenna at the slit of an SRR, using the same settings employed in the simulations, to excite MSPs on the metasurface. A split loop with a radius of 4 mm that serves as a magnetic dipole antenna is located in the *xy* plane 2 mm above the metasurface. The magnetic dipole antenna is fixed on the arm of a 3D movement platform, which moves in the *xy* plane point to point, with a 4 mm step. By connecting both of the antennas with a vector network analyzer, we obtain the distributions of the *H_z_* field over the metasurface, as shown in **Figure** **4**f–j.

**Figure 4:**
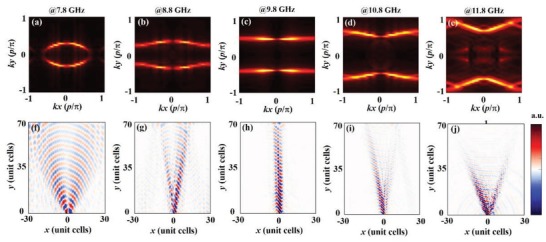
Experimental observation of a transition of the EFCs and nondiffracting phenomenon on the MHMS. The upper row presents first‐band EFCs of the present MHMS in the first Brillouin zone at a) 7.8 GHz, b) 8.8 GHz, c) 9.8 GHz, d) 10.8 GHz, and e) 11.8 GHz. The lower row presents *H_z_* field distributions at a plane 2 mm above the metasurface that was excited by an electric dipole at the slit of an SRR at f) 7.8 GHz, g) 8.8 GHz, h) 9.8 GHz, i) 10.8 GHz, and j) 11.8 GHz. We can clearly see normal diffraction ((f)–(g)), nondiffraction (h), and anomalous diffraction (i)–(j) as the EFCs change from elliptical to flat and then to hyperbolic.

We can see that the measured *H_z_* field distributions are in excellent agreement with the simulated distributions, i.e., the wavefronts of the MSPs change from convex to flat and then to concave. By applying a 2D spatial Fourier transformation of the magnetic field distributions, we obtain the EFCs of the dispersions of the fabricated metasurface at various frequencies (Figure 4a–e). Note that due to the mirror symmetry and the time‐reversal symmetry preserved in the present metasurface, the EFCs should be mirror‐symmetric in both *x* and *y* directions. Therefore, when calculating EFCs from real‐space experimental images, we employ the mirror symmetry operator to make the EFCs symmetric. The measured EFCs are almost the same as those in the simulations, i.e., the shapes of the EFCs vary from a closed ellipse to flat lines and eventually to an open hyperbola. Interestingly, at the transition point, the MSPs propagate in a nondiffracting manner due to the flat EFC. This self‐collimation phenomenon^40^ has potential applications in planar magnifying hyperlenses. Additionally, we directly observe anomalous diffraction of the MSPs at frequencies of 10.8 and 11.8 GHz.

One of the most interesting phenomena observed for the present metasurface is negative refraction in the hyperbolic regime.^41^, ^42^ In the experiment, an electric dipole serves as the point source/image at one boundary of the metasurface and will excite the MSPs, which refocus in the air at the opposite side of the metasurface (**Figure** **5**a). In Figure 5b, we can see the negative refraction of the energy flow at the interface between the metasurface and air. This result can be explained by the isofrequency contour of the metasurface and air, as shown in Figure 5c. All incoming wavevectors are included within the EFCs of the MHMS, enabling all‐angle negative refraction.^18^, ^43^ This phenomenon can be applied to the design of planar focusing and imaging devices.

**Figure 5:**
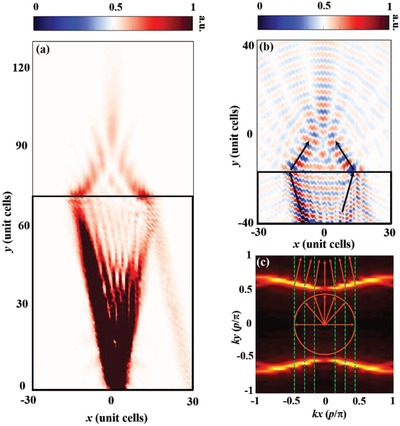
Experimental observation of all‐angle negative refraction and focusing of MSPs in the hyperbolic regime. a) Measured magnetic energy distributions in the *xy* plane 2 mm above the metasurface at 11.0 GHz. b) Measured *H_z_* field distributions in the *xy* plane 2 mm above the present metasurface at 11.0 GHz. The solid black rectangles represent the MHMS; the arrows represent the energy flow. c) Isofrequency contour of the metasurface and air at 11.0 GHz. The orange circle is an isofrequency contour of the air. The orange arrows represent the propagation direction of the energy flow. When the EM wave propagates from air to the MHMS, all‐angle negative refraction will occur at the interface between air and the MHMS, which also results in focusing when the EM energy leaves the metasurface.

It has been reported that SPs on a hyperbolic metasurface show strong spatial localization.^23^ Here, instead of SPs, we experimentally demonstrate such behavior of MSPs on the present MHMS, as shown in **Figure** **6**a–d. Additionally, we find that the spatial localization of the MSPs is not only strong but also frequency‐dependent, i.e., the opening angle of the MSP cone becomes larger as the frequency increases. This phenomenon has a potential impact on the development of spatial multiplexers.

**Figure 6:**
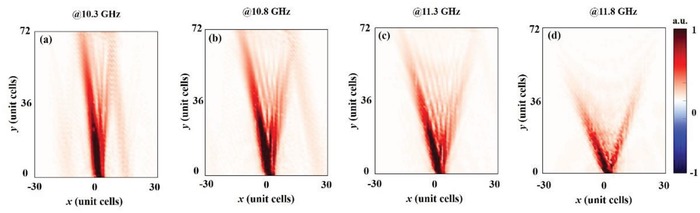
Experimental observation of frequency‐dependent strong spatial localization of MSPs on the MHMS. a–d) Distributions of the measured amplitude of the magnetic field in the *xy* plane 2 mm above the metasurface at a) 10.3 GHz, b) 10.8 GHz, c) 11.3 GHz, and d) 11.8 GHz. The EM pattern is excited by an electric dipole moment at the slit of an SRR.

In summary, we propose the concept of an MHMS on which MSPs travel as a divergent beam. Physically, the MSPs arise from anisotropic magnetic excitations, stemming from coupled magnetic dipoles that are provided, in practice, by circular surface/displacement currents. As a proof of concept, we designed and fabricated an ultrathin MHMS that consists of arrays of double‐slit SRRs. We directly observed a transition of the EFCs in momentum space, diffraction‐free propagation, anomalous diffraction, all‐angle negative refraction, and frequency‐dependent strong spatial distributions of the MSPs on the metasurface. The MHMS has the advantages of ultrathin thickness, easy access, and processing, light weight, rich functionalities, and so forth. These metasurfaces could be used to manipulate MSPs with strong confinement and could be applied in the design of planarized focusing and imaging devices and spatial multiplexers. Although this concept is verified in the microwave band, its universality enables it to be applied even at terahertz and optical frequencies, which facilitates the design of novel optical devices with strong magnetic light–matter interactions in quantum optics. Additionally, this uniaxial metasurface can be applied as a cornerstone for on‐chip transformation optics‐based devices.

## Conflict of Interest

The authors declare no conflict of interest.

## Supporting information

Supplementary
