# Supplementary material for: Magnetic Hyperbolic Metasurface: Concept, Design, and Applications
Source: Adv Sci (Weinh). 2018 Nov 12;5(12):1801495. doi: 10.1002/advs.201801495 (PMC6299717; doi:10.1002/advs.201801495)
Supplement: Supplementary file 1 — Supplementary [file ADVS-5-1801495-s001.pdf]

Copyright WILEY-VCH Verlag GmbH & Co. KGaA, 69469 Weinheim, Germany, 2018.

ADVANCED SCIENCE
Open Access

Supporting Information

for *Adv. Sci.,* DOI: 10.1002/advs.201801495

Magnetic Hyperbolic Metasurface: Concept, Design, and Applications

Yihao Yang, Pengfei Qin, Bin Zheng, Lian Shen, Huaping Wang, Zuojia Wang, Erping Li, Ranjan Singh, and Hongsheng Chen**

Supporting Information:
Magnetic Hyperbolic Metasurface: Concept, Design and Applications

Yihao Yang[1,2,3,4], Pengfei Qin[2], Bin Zheng[1,2], Lian Shen[1,2], Huaping Wang[5], Zuojia Wang[6], Erping Li[2], Ranjan Singh[3,4] and Hongsheng Chen[1,2,*]

[1]State Key Laboratory of Modern Optical Instrumentation and The Electromagnetics Academy at Zhejiang University, Zhejiang University, Hangzhou 310027, China.

[2]Key Laboratory of Micro-Nano Electronics and Smart System of Zhejiang Province, College of Information Science and Electronic Engineering, Zhejiang University, Hangzhou 310027, China.

[3]Division of Physics and Applied Physics, School of Physical and Mathematical Sciences, Nanyang Technological University, 21 Nanyang Link, Singapore 637371, Singapore.

[4]Centre for Disruptive Photonic Technologies, The Photonics Institute, Nanyang Technological University, 50 Nanyang Avenue, Singapore 639798, Singapore.

[5]Institute of Marine Electronics Engineering, Ocean College, Zhejiang University, Hangzhou 310058, China.

[6]School of Information Science and Engineering, Shandong University, Jinan 250100, China.

[*]hansomchen@zju.edu.cn

1. Equivalent circuit models

Obviously, due to lacking C4 symmetry, the unit cell of the magnetic hyperbolic metasurface exhibits different responses when electromagnetic waves propagate along the x and y directions, respectively. To better understand the underlying mechanism, we consider equivalent circuit models in which magnetic surface plasmons (MSPs) propagate along the x (Fig. S1(a)) and y directions (Fig. S1(b)). Interestingly, the mutual capacitances, $C_2\,(C_2\,')$, do not affect the MSP frequencies which is $f_x = 1/\sqrt{LC_1}$ and $f_y = 1/\sqrt{L'C_1\,'}$ when the in-plane wavevector is along the x and y directions, respectively. Here, $C_1\,(C_1\,')$ and $L\,(L')$ are the capacitance and inductance inside the unit cell, respectively. From the electric field distributions, one can see that the electric field localizes at different regions when the MSP propagates along x and y directions, respectively. Therefore, the induced capacitances and inductances are different, resulting in the different MSP frequencies. In our case, $f_x < f_y$. When $f_0 < f_x < f_y$ (f_0 is the operational frequency), the MSPs propagate along both orthogonal directions, and the EFCs are elliptical. Alternatively, when $f_x < f_0 < f_y$, only the MSPS along the y direction exist. By altering the geometries appropriately, one can tailor the EFCs to be hyperbolic.

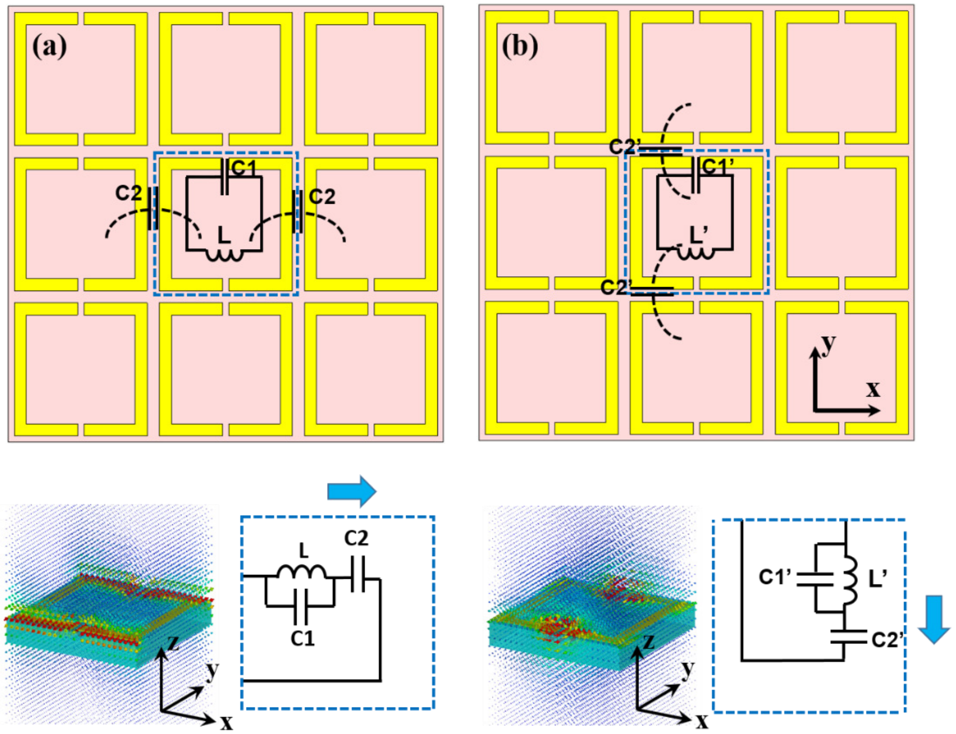

Figure S1. (a)-(b) Equivalent circuit models when electromagnetic waves propagate along the x and y directions, respectively. The inset at the left bottom of each figure represents the electric field distributions when $k_x = \pi / p, k_y = 0$ and $k_x = 0, k_y = \pi / p$, respectively.

2. Calculation of power of different multipoles

To calculate the localized EM modes in the hyperbolic metasurface, the eigenmode solver of the commercially available software, Computer Simulation Technology (CST) Microwave Studio is employed. With the extracted surface current, the moments of electric dipole, magnetic dipole, electric quadrupole and magnetic quadrupole are calculated according to multipole scattering theory [S1]:

electric dipole moment:

$$\vec{p} = \frac{1}{i\omega}\int d^3r\,\vec{j}, \tag{1}$$

magnetic dipole moment:

$$\vec{m} = \frac{1}{2c}\int d^3r\left(\vec{r}\times\vec{j}\right), \tag{2}$$

electric quadrupole moment:

$$Q_{\alpha\beta} = \frac{1}{i2\omega}\int d^3r\left[r_\alpha j_\beta + r_\beta j_\alpha - \frac{2}{3}\delta_{\alpha\beta}\left(r\cdot j\right)\right], \tag{3}$$

magnetic quadrupole moment:

$$M_{\alpha\beta} = \frac{1}{3c}\int d^3r\left[\left(\vec{r}\times\vec{j}\right)_\alpha r_\beta + \left(\vec{r}\times\vec{j}\right)_\beta r_\alpha\right], \tag{4}$$

where **r** is distance vector from the origin to point (x, y, z) in a Cartesian coordinate system; **j** is current density at the point r (x, y, z); c is the speed of light in the vacuum; and $\alpha,\beta = x, y$. The **r** and **j** are exported from CST software at different frequencies. Then we can get the far-field scattering power of each multipole

$$I_p = \frac{2\omega^4}{3c^3}\left|\vec{p}_z\right|^2, \tag{5}$$

$$I_m = \frac{2\omega^4}{3c^3}\left|\vec{m}_{xy}\right|^2, \tag{6}$$

$$I_Q = \frac{\omega^6}{5c^5}Q_{\alpha\beta}Q_{\alpha\beta}, \tag{7}$$

$$I_M = \frac{\omega^6}{20c^5}M_{\alpha\beta}M_{\alpha\beta}, \tag{8}$$

which is shown in Fig. S2.

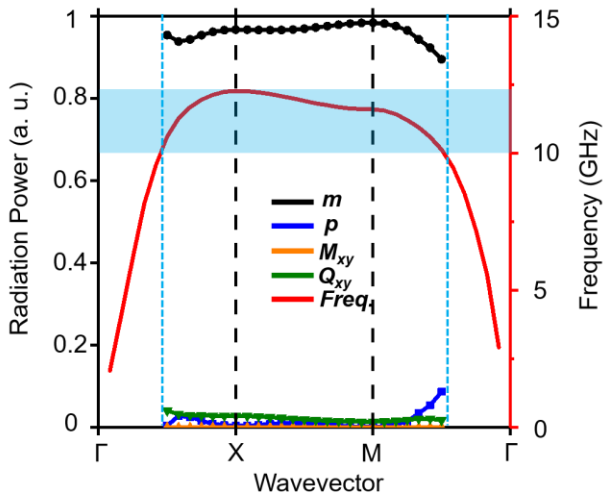

Figure S2. Numerical calculations of normalized radiation power of moments of electric dipole (p), magnetic dipole (m), electric quadrupole (M_{xy}), and magnetic quadrupole (Q_{xy}) in the hyperbolic regime, respectively. The red line represents the dispersion of the hyperbolic metasurface. The region highlighted in blue is the hyperbolic regime.

References

[S1]E. E. Radescu, G. Vaman, Phys. Rev. E **2002**, 65, 046609.
